# AgriPV system with climate, water and light spectrum control for safe, healthier and improved crop production

**DOI:** 10.12688/openreseurope.18876.1

**Published:** 2024-12-18

**Authors:** Miray Çelikbilek Ersundu, Ali Erçin Ersundu, Colin Osborne, Maria Ruiz

**Affiliations:** 1 Faculty of Chemical and Metallurgical Engineering, Department of Metallurgical and Materials Engineering, Glass Research and Development Laboratory, Yildiz Technical University, Istanbul, 34220, Turkey; 2 School of Biosciences, Alfred Denny Building, The University of Sheffield, Sheffield, England, S10 2TN, UK; 3 Innovation, Avenida de Manoteras, R2M Solution Spain, Madrid, Spain, 28050, Spain

**Keywords:** Agrivoltaics; renewable energy; nanoparticle technology; sustainable agriculture; crop health; circular economy; light spectrum control; climate adaptability

## Abstract

The PV4Plants project aims to optimise the synergy between agriculture and photovoltaic (agriPV) systems to improve crop yield, land use efficiency, and renewable energy generation. Using cutting-edge nanoparticles coated between PV panels to optimise light transmission, the system tailors conditions for specific crops and climatic regions. Demonstrations in Türkiye, Spain, and Denmark test the adaptability and effectiveness of these systems, to evaluate improvements in crop health and renewable energy output. Central to the project are efforts to increase recyclability and promote farmer engagement through innovative financing models and policy recommendations.

## Introduction

### The growing need for sustainable AgriPV systems in agriculture and energy

The agricultural sector is a significant contributor to greenhouse gas (GHG) emissions, accounting for approximately 10% of all GHG emissions in the EU, a percentage that is set to increase as other sectors rapidly decarbonise. As part of European efforts to meet ambitious climate targets, such as those outlined in the Farm-to-Fork strategy and the Common Agricultural Policy (CAP), decarbonizing agriculture has become a primary objective. The sector’s heavy reliance on fossil fuels for energy—used in heating, cooling, irrigation, and appliances like large refrigerators—poses serious environmental challenges. This dependence on fossil-based energy, coupled with the constant pressure on farmers to increase productivity, creates a cycle that both harms the environment and discourages innovation. 

Farmers often face a dilemma: adopting innovative technologies can carry the risk of uncertain yields, which in turn makes them hesitant to experiment. On the other hand, increasing agricultural production often leads to higher energy consumption, frequently in the form of inefficient power systems such as diesel-powered generators, which further intensifies the use of fossil fuels. Additionally, renewable energy technologies, like solar and wind power, are often seen as competitors for arable land, a precious resource in farming. Therefore, solutions that address both the energy needs of agriculture and land-use efficiency are critical if Europe is to meet its Green New Deal targets, which include the goal of full decarbonization by 2050.

### AgriPV: a dual-use solution

One promising approach is agrivoltaics (also known as AgriPV), a technology that integrates photovoltaic (PV) energy generation directly into agricultural fields. According to DIN SPEC 91434, "Agrovoltaics is the combined use of the same land area for agricultural production as the primary use and for photovoltaic electricity production as a secondary use." AgriPV presents a strong opportunity to minimise the agricultural sector’s reliance on fossil fuels while simultaneously boosting the efficiency of land use. This dual-use technology offers several key advantages:

1. **Improved land efficiency**: AgriPV avoids the competition for land between agriculture and energy production, utilising the same area for both purposes.
2. **Renewable local energy generation**: AgriPV provides farmers with locally generated, renewable energy that can be consumed directly, minimising energy transmission losses, reducing dependency on external energy sources, and helping to alleviate energy poverty.
3. **Social and financial opportunities**: AgriPV creates new revenue streams for farmers, offering higher turnover per hectare of cultivated land, new income potentials, and the creation of rural jobs, which can help combat the depopulation of rural areas

In addition to these economic and environmental benefits, AgriPV systems offer direct advantages for agricultural productivity. The shading provided by PV panels can create a favourable microclimate beneath the structures, mitigating extreme temperatures, reducing evapotranspiration, and protecting crops from excessive sunlight, heatwaves, rainstorms, high winds, and hail. These protective qualities allow for more resilient agricultural practices, particularly in arid regions.

However, several challenges must be addressed for AgriPV to fully realise its potential as a game-changer for agriculture and renewable energy. These challenges include the need for technological adaptability to different climates and crop types, cost affordability, and the acceptance of these innovations by end-users like farmers. Cooperation among stakeholders, including policymakers, farmers, and renewable energy developers, is crucial. Additionally, increased circularity, new business models, and supportive policies and regulations are needed to facilitate the widespread adoption of AgriPV technology ^1– 8^.

### Objectives of PV4Plants

The overarching goal of PV4Plants is to improve the synergy between agricultural production and photovoltaic (PV) energy generation, maximising land-use efficiency. The specific objectives of the project include:

**1. Optimise light spectrum**: Through the application of glass color converter (GCC) materials between PV panels, PV4Plants aims to adjust the light spectrum for enhanced photosynthetic activity, targeting specific wavelengths that support crop growth without comprising energy production.
**2. Increase crop yield and health:** The system improves growing conditions beneath PV panels by controlling key variables such as light, water, and microclimate conditions. The focus is on healthy, high-yield crops, with real-time monitoring to adjust conditions dynamically.
**3. Sustainability and circular economy:** A key priority is to ensure the system is sustainable. This includes designing PV components for recyclability, promoting reuse, and certifying the system under the **Environmental Product Declaration (EPD)** and **ISO 14021** standards.
**4. Boost renewable energy generation** through an improved PV panel design that adapts to agricultural needs without compromising energy output.
**5. Demonstrate scalability and replicability:** Testing and validating the PV4Plants system in diverse climatic regions with different crops ensures that the technology is adaptable and scalable across Europe.
**6. Stakeholder engagement and financial sustainability:** Through innovative engagement strategies with farmers and policy recommendations, the project seeks to boost trust in AgriPV technologies and provide models for financing and scaling the system in rural and agricultural communities.

These goals align with broader European Union objectives of achieving a sustainable, secure, and competitive energy supply while enhancing agricultural productivity.

## System design, implementation, and testing

### Advanced system design

**Figure 1.**
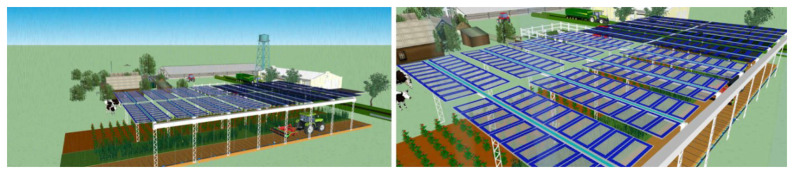
A schematic of the AgriPV system (left). Panels with regular and GCC glass on an agriPV system (right).

PV4Plants integrates an innovative nanoparticle-enhanced PV system ( Figure 1) designed to optimise light transmission for both crop growth and energy generation. The system features:

- **Nanoparticle-coated PV panels:** The PV panels are coated with **perovskite-based glass nanocomposites,** which modify the light spectrum. These materials enhance red light transmittance, which is crucial for plant photosynthesis, while reducing UV, blue, and green light transmittance and maintaining or improving solar energy conversion efficiency.
- **Rainwater harvesting:** The system incorporates rainwater collection features, especially vital for arid regions. This function ensures efficient water use for irrigation and minimises the reliance on external water sources.
- **Proactive microclimate control:** PV4Plants employs a real-time monitoring and control system, using sensors to track soil moisture, air temperature, humidity and light intensity. This system dynamically adjusts the microclimate under the PV panels, including panel tilt and irrigation levels, optimising both crop conditions and energy production.

### Real-time monitoring and proactive control

The system’ **s real-time monitoring and dynamic control** is one of its most innovative aspects. Data collected from a range of sensors (light, temperature, humidity, soil moisture) are processed to make proactive adjustments to the environment. For example, during hot weather, panels may be adjusted to increase shading, reducing plant stress and improving water use efficiency. Conversely, during low-light conditions, panel angles are adjusted to increase light penetration. This dynamic approach not only enhances agricultural outcomes but also ensures the PV system’s energy output remains high.

### Demonstration sites and testing

**Figure 2.**
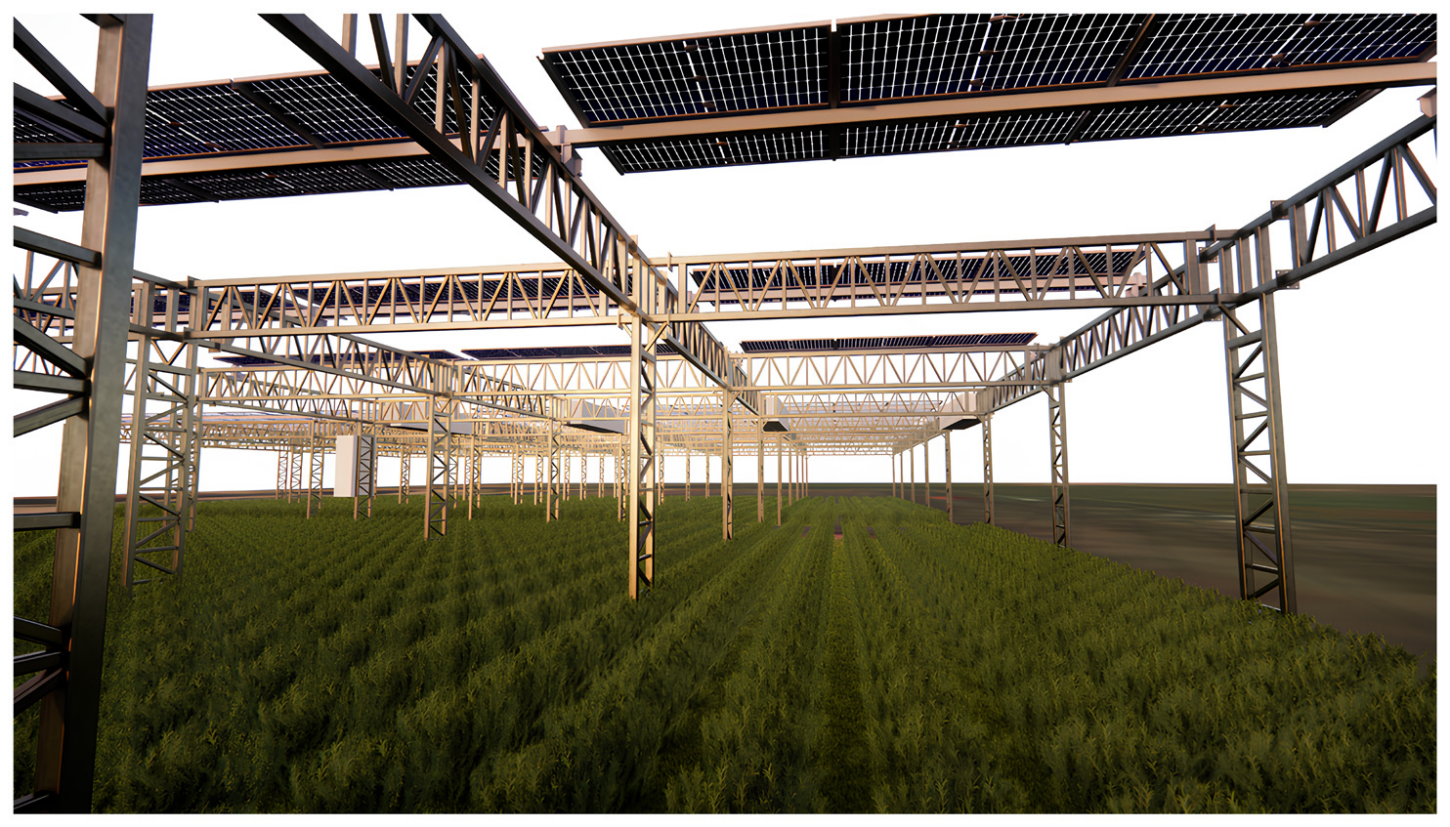
Crop field with PV4Plants technology in Bursa.

**Figure 3.**
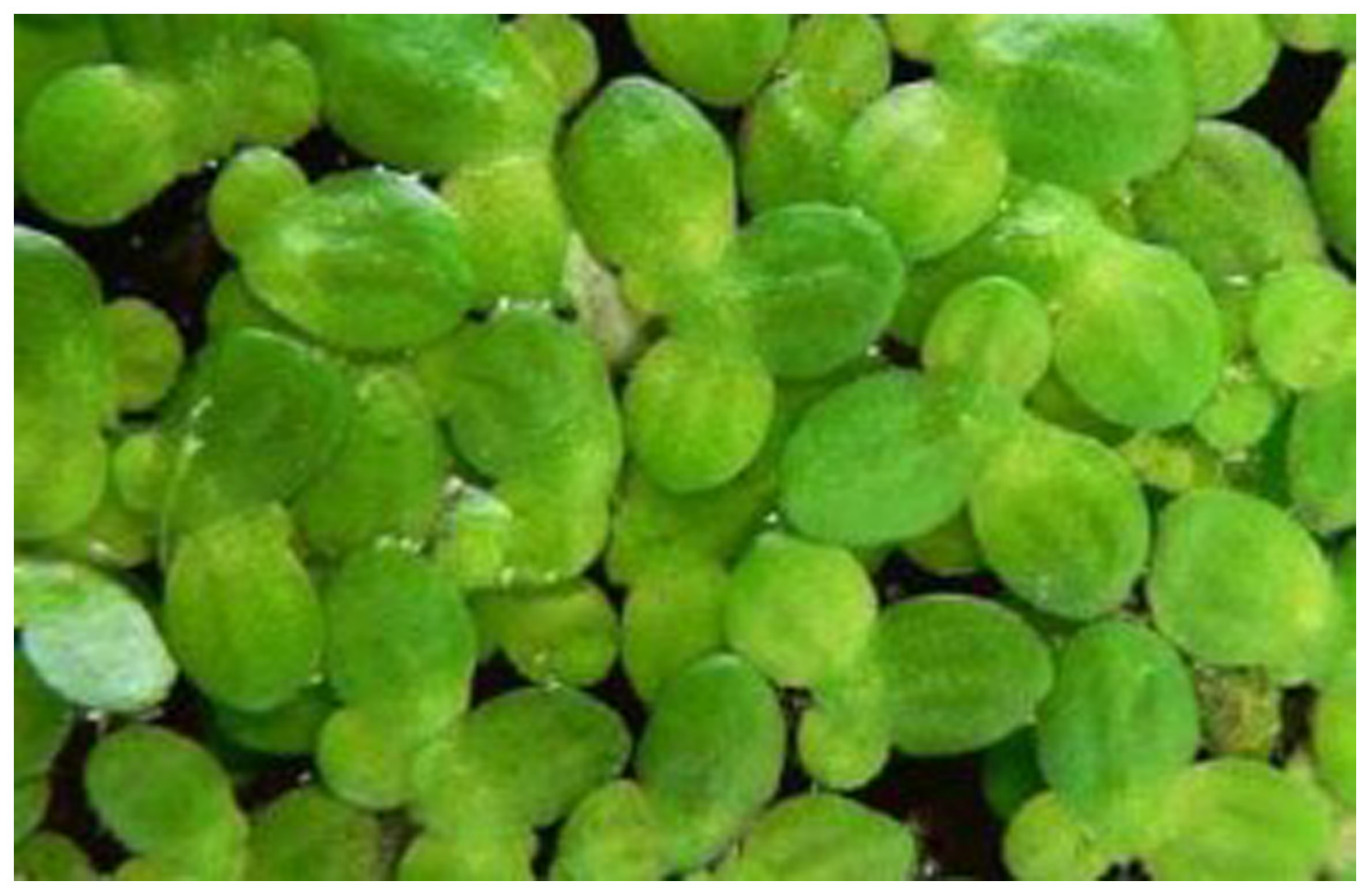
Microalgae that will be cultivated in Avila.

PV4Plants is being piloted at three geographically and climatically diverse locations to test its adaptability and scalability:

- **Bursa, Türkiye** ( Figure 2) **:** A semi-arid region, focusing on crops like tomatoes and green peas. This site tests the system’s efficiency in hot, dry conditions while utilising the rainwater harvesting mechanism.
- **Avila, Spain:** Focusing on microalgae production ( Figure 3), this site evaluates the system’s adaptability in a Mediterranean climate and its ability to support non-traditional crops under agriPV systems.
- **Hoje Taastrup, Denmark:** In this northern European climate, leafy vegetables such as cress and spinach are grown, testing the system’s capability to function in cooler, wetter conditions.

Each site incorporates local stakeholder engagement and policy alignment to ensure that the technology addresses specific regional needs, increasing the likelihood of adoption and scalability.

### Material innovation and light spectrum optimisation

A key aspect of the PV4Plants system is the **nanoparticle-based light spectrum tuning.** The use of **perovskite-based glass nanocomposites** allows for the customisation of light conditions beneath the PV panels, optimising the photosynthetically active radiation (PAR) range. This enables the system to increase light intensity for photosynthesis while maintaining or enhancing the PV efficiency, particularly during the peak growth period of crops. For example, tomato plants thrive under red light, so the panels are turned to maximise red light transmission while ensuring energy generation.

### Multi-criteria assessment and financial models

The project incorporates a **multi-criteria assessment** methodology to evaluate its impact across environmental, economic, and social factors. In each demonstration site, the following metrics are tracked:

- **Energy generation:** Energy efficiency and total energy output from the PV panels.
- **Crop yield and health:** Crop productivity and plant health metrics compared to control fields.
- **Water efficiency:** Water usage per crop and efficiency improvements due to the rainwater harvesting system.
- **Environmental impact:** Recyclability of PV materials and lifecycle assessments to evaluate the system’s overall environmental footprint.

Additionally, the project explores innovative financing and business models, ensuring that the system is not only effective but also financially viable for farmers and investors. Policy recommendations are also being developed in collaboration with public authorities to support the broader adoption of AgriPV systems across Europe.

## Results and analysis

### Crop performance and yield improvement

Preliminary results come from lab testing of the impacts of spectral tuning on crop performance. Tomatoes grown under red enrichment had lower rates of photosynthesis, although growth of the plants was unaffected. However, initial measurements suggest an improvement in fruit quality.

### Energy generation and efficiency

The application of **spectrally tunable materials** to the PV panels has demonstrated that the system can maintain high energy output while optimising light transmission for crops.

### Water conservation

Thermal imaging from indoor trials mimicking the conditions in Türkiye showed that crops grown under the system’s red light optimisation had higher leaf temperatures, suggesting reduced transpiration and water savings. This is particularly important for regions facing water scarcity.

### Sustainability and circular economy

**Figure 4.**
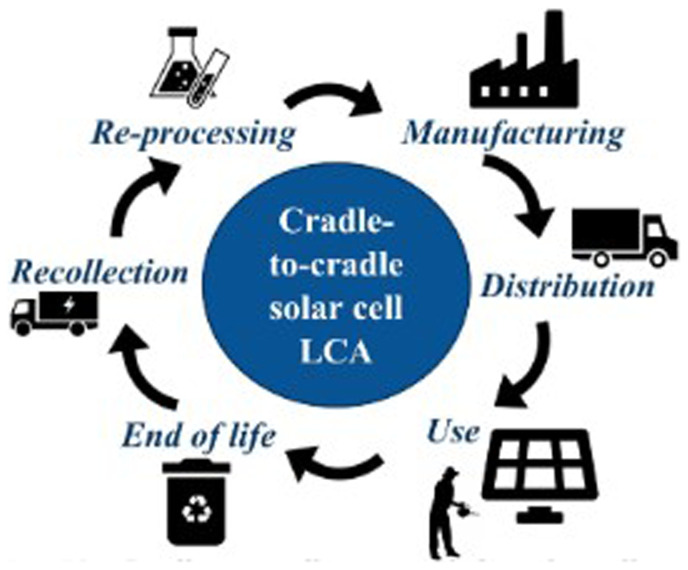
Cradle-to-cradle approach.

PV4Plants incorporates a strong emphasis on sustainability, from the design phase to the end-of-life cycle of the system. All materials used in the PV panels are designed to be recyclable, and the project is working toward certification under **Environmental Product Declarations (EPD)** and **ISO 14021** standards. The system’s life cycle analysis indicates that PV4Plants reduces environmental impact by using recycled materials in panel construction and optimising water use through rainwater harvesting as well as using a cradle-to-cradle approach ( Figure 4) for luminescent GCC panels.

### Stakeholder engagement and social impact

A significant part of PV4Plants’ success lies in its engagement with local stakeholders, including farmers, public authorities, and NGOs. Early-stage feedback from farmers at the Turkish and Spanish sites indicates a high level of acceptance, with enthusiasm for the water-saving features and the potential for increased crop yields. Moreover, the development of tailored policy recommendations will facilitate the broader adoption of AgriPV systems, contributing to rural development and sustainability goals.

## Evaluation of results and broader implications

PV4Plants’ early trial results confirm the potential of AgriPV systems to simultaneously improve agricultural productivity and generate renewable energy. The optimised light spectrum, coupled with real-time environmental adjustments, could prove effective in enhancing crop yield, particularly in climate-sensitive crops in arid regions. This dual benefit suggest the future viability of AgriPV systems in regions with diverse climates, where both agricultural output and energy generation are priorities.

The project’s sustainability efforts—focusing on recyclability, water conservation, and life cycle impact reduction—make PV4Plants a model for future AgriPV systems. The inclusion of rainwater harvesting systems further highlights the system’s potential for water-scarce regions. Moreover, the positive response from stakeholders and local farmers suggests that PV4Plants can be widely adopted, provided sufficient policy support and financial mechanisms are in place.

## Conclusion and future directions

PV4Plants offers a groundbreaking solution to one of the most pressing challenges of modern agriculture: the need to balance food production with renewable energy generation on limited land. Through its innovative use of nanoparticle-enhanced PV panels, real-time monitoring systems, and sustainability-driven design, the project presents a scalable model for AgriPV systems that can be adapted to different climates and crop varieties.

Future directions for the project will include further refinement of the light spectrum optimization for additional crops and continued expansion of the system to other regions. The next phase will also focus on developing long-term financial models and working closely with policymakers to create supportive frameworks for large-scale implementation.

## Ethics and consent

After completion of the Horizon Europe proposal ethics self-assessment, no ethics issues were identified, therefore ethics approval is not required.

## Disclaimer

The views expressed in this article are those of the author(s). Publication in ORE does not imply endorsement by the European Commission.

## Data Availability

No data is associated with this article.
