# Supplementary material for: AgriPV system with climate, water and light spectrum control for safe, healthier and improved crop production
Source: Open Res Eur. 2025 Mar 6;4:267. Originally published 2024 Dec 18. [Version 2] doi: 10.12688/openreseurope.18876.2 (PMC11950720; doi:10.12688/openreseurope.18876.2)
Supplement: Peer review history

# Reviewer report: Reviewer response for version 2

**DOI:** 10.21956/openreseurope.21373.r51850

**Published:** 2025-03-31

**Authors:** Sharon Hilarydoss

**Affiliations:** 1 Department of Mechanical Engineering, Indian Institute of Petroleum and Energy (IIPE), Visakhapatnam, Andhra Pradesh, India

My comments have been addressed

Where applicable, are recommendations and next steps explained clearly for others to follow? (Please consider whether others in the research community would be able to implement guidelines or recommendations and/or constructively engage in the debate)

Yes

Does the article adequately reference differing views and opinions?

Partly

Are all factual statements correct, and are statements and arguments made adequately supported by citations?

Yes

Is the rationale for the Open Letter provided in sufficient detail? (Please consider whether existing challenges in the field are outlined clearly and whether the purpose of the letter is explained)

Partly

Is the Open Letter written in accessible language? (Please consider whether all subject-specific terms, concepts and abbreviations are explained)

Yes

Reviewer Expertise:

SOLAR ENERGY, DESALINATION, PV-WATER SYNERGY, ALTERNATE FULES

I confirm that I have read this submission and believe that I have an appropriate level of expertise to confirm that it is of an acceptable scientific standard.

# Reviewer report: Reviewer response for version 2

**DOI:** 10.21956/openreseurope.21373.r51977

**Published:** 2025-03-27

**Authors:** Meita Rumbayan

**Affiliations:** 1 Sam Ratulangi University, Manado, Indonesia

The article partly explains recommendations and next steps clearly, but there is room for improvement. Here's the evaluation:

 Strengths:

1. Policy Recommendations: The article outlines plans to develop a regulatory framework for AgriPV adoption, including guidelines on land use, financial incentives, and stakeholder inclusion (e.g., *"The project will provide clear guidelines on AgriPV design... and propose strategies for financial incentives"*).
2. Future Directions: It mentions refining light spectrum optimization for additional crops, expanding to new regions, and developing long-term financial models, which provide a roadmap for further research.
3. Stakeholder Engagement: The project’s approach to involving farmers, policymakers, and local communities (e.g., roundtables, workshops) offers a replicable model for similar initiatives.

Weaknesses:

1. Lack of Specificity: While the article mentions policy recommendations, it does not detail actionable steps (e.g., specific policy changes or legislative proposals).
2. No Clear Guidelines for Replication: The technical setup (e.g., panel configurations, sensor systems) is described, but exact parameters (e.g., optimal light spectra for crops, tilt adjustments) are not provided, making it difficult for others to replicate the system.
3. Missing Quantitative Benchmarks: The article lacks measurable targets (e.g., "increase crop yield by X%") or performance thresholds, which would help others assess feasibility.

Conclusion: Partly

 The recommendations are broadly outlined but lack granularity for direct implementation. The next steps are directionally clear (e.g., scalability, policy work) but would benefit from concrete, actionable details.

 Suggestions for Improvement:

- Include specific policy proposals (e.g., subsidy structures, zoning laws).
- Provide technical guidelines (e.g., panel-to-crop ratios for different climates).
- Define success metrics (e.g., energy output thresholds, crop yield improvements).
- Add a timeline for future phases (e.g., pilot expansions, policy advocacy milestones).

This would better enable the research community to engage constructively or replicate the work.

Where applicable, are recommendations and next steps explained clearly for others to follow? (Please consider whether others in the research community would be able to implement guidelines or recommendations and/or constructively engage in the debate)

Partly

Does the article adequately reference differing views and opinions?

Yes

Are all factual statements correct, and are statements and arguments made adequately supported by citations?

Yes

Is the rationale for the Open Letter provided in sufficient detail? (Please consider whether existing challenges in the field are outlined clearly and whether the purpose of the letter is explained)

Yes

Is the Open Letter written in accessible language? (Please consider whether all subject-specific terms, concepts and abbreviations are explained)

Yes

Reviewer Expertise:

Renewable energy

I confirm that I have read this submission and believe that I have an appropriate level of expertise to confirm that it is of an acceptable scientific standard, however I have significant reservations, as outlined above.

# Reviewer report: Reviewer response for version 2

**DOI:** 10.21956/openreseurope.21373.r51851

**Published:** 2025-03-18

**Authors:** Silvia Ma Lu

**Affiliations:** 1 Mälardalen University, Västerås, Sweden

The authors have revised the open letter in response to the comments on the previous version and have incorporated several of the suggested improvements. The revised version aligns more closely with the intended format of an open letter, and I have no further comments. I look forward to seeing the results of this very interesting project in the future.

Where applicable, are recommendations and next steps explained clearly for others to follow? (Please consider whether others in the research community would be able to implement guidelines or recommendations and/or constructively engage in the debate)

Not applicable

Does the article adequately reference differing views and opinions?

Yes

Are all factual statements correct, and are statements and arguments made adequately supported by citations?

Partly

Is the rationale for the Open Letter provided in sufficient detail? (Please consider whether existing challenges in the field are outlined clearly and whether the purpose of the letter is explained)

Partly

Is the Open Letter written in accessible language? (Please consider whether all subject-specific terms, concepts and abbreviations are explained)

Yes

Reviewer Expertise:

Agrivoltaics, Solar Energy, Spectral Irradiance, Wavelength-Selective Solar Photovoltaic

I confirm that I have read this submission and believe that I have an appropriate level of expertise to confirm that it is of an acceptable scientific standard.

# Reviewer report: Reviewer response for version 1

**DOI:** 10.21956/openreseurope.20426.r49421

**Published:** 2025-01-27

**Authors:** Sharon Hilarydoss

**Affiliations:** 1 Department of Mechanical Engineering, Indian Institute of Petroleum and Energy (IIPE), Visakhapatnam, Andhra Pradesh, India

Include quantitative results also in abstract

 “Through the application of glass color converter (GCC) materials between PV panels” Why gap is required between panels? Tinted semi-transparent solar modules can be used? Give your opinion and discussion on this

 “diverse climatic regions” What are climatic conditions considered?

 “For example, during hot weather, panels may be adjusted to increase shading” Does it not affect PV performance? Is there an optimum trade-off between PV performance and crop yield?

 “This dynamic approach not only enhances agricultural outcomes but also ensures the PV system’s energy output remains high”. How this is possible if PV is tilted from its optimum position? Clarify this

 Quantitative results are missing all over. Include them

 Farming environment will be mostly dusty. What’s the plan to handle dust deposit?

 Include quantitative results also in abstract

 “Through the application of glass color converter (GCC) materials between PV panels” Why gap is required between panels? Tinted semi-transparent solar modules can be used? Give your opinion and discussion on this

 “diverse climatic regions” What are climatic conditions considered?

 “For example, during hot weather, panels may be adjusted to increase shading” Does it not affect PV performance? Is there an optimum trade-off between PV performance and crop yield?

 “This dynamic approach not only enhances agricultural outcomes but also ensures the PV system’s energy output remains high”. How this is possible if PV is tilted from its optimum position? Clarify this

 Quantitative results are missing all over. Include them

 Farming environment will be mostly dusty. What’s the plan to handle dust deposit?

Where applicable, are recommendations and next steps explained clearly for others to follow? (Please consider whether others in the research community would be able to implement guidelines or recommendations and/or constructively engage in the debate)

Yes

Does the article adequately reference differing views and opinions?

Partly

Are all factual statements correct, and are statements and arguments made adequately supported by citations?

Yes

Is the rationale for the Open Letter provided in sufficient detail? (Please consider whether existing challenges in the field are outlined clearly and whether the purpose of the letter is explained)

Partly

Is the Open Letter written in accessible language? (Please consider whether all subject-specific terms, concepts and abbreviations are explained)

Yes

Reviewer Expertise:

SOLAR ENERGY, DESALINATION, PV-WATER SYNERGY, ALTERNATE FULES

I confirm that I have read this submission and believe that I have an appropriate level of expertise to confirm that it is of an acceptable scientific standard, however I have significant reservations, as outlined above.

# Response

**Published:** 2025-02-17

**Authors:** María Ruiz

**Affiliations:** Innovation, Not Applicable, Madrid, Spain, Spain

**Through the application of glass color converter (GCC) materials between PV panels” Why gap is required between panels? Tinted semi-transparent solar modules can be used? Give your opinion and discussion on this “diverse climatic regions” What are climatic conditions considered?**

 The gap between panels is necessary to achieve an optimal light-to-electricity output ratio for the designed GCC panels. GCC panels are highly transmissive, allowing light to pass through and reach the crops beneath the agrivoltaic system. In contrast, conventional Si solar cells are opaque and do not permit light transmission. Therefore, the design intentionally incorporates gaps between GCC panels and Si solar cells to balance energy generation and agricultural productivity.While tinted semi-transparent solar modules can be used, the current innovation action project is designed around standard Si solar cells due to their widespread use and large-scale production, making them the most accessible and commercially viable option at present. “Diverse climatic regions” What are climatic conditions considered.

**“For example, during hot weather, panels may be adjusted to increase shading” Does it not affect PV performance? Is there an optimum trade-off between PV performance and crop yield?**

 By "diverse climatic regions," we are not referring to seasonal weather changes but rather to the different geographic locations where the demonstration sites will be installed—Türkiye, Spain, and Denmark. While Türkiye and Spain share a warm Mediterranean climate, Denmark has a completely different climate. Therefore, the term specifically highlights the variation in environmental conditions across these locations, where the novel GCC agrivoltaic sites will be implemented. Regarding the second question, yes, there is an optimal trade-off between PV performance and crop yield. This balance will be determined through data collection and analysis after the installation of the demonstration sites. The impact of panel adjustments on both shading and PV efficiency will be evaluated for different crops in various geographic regions to find the most effective configuration.

**“This dynamic approach not only enhances agricultural outcomes but also ensures the PV system’s energy output remains high”. How is this possible if PV is tilted from its optimum position? Clarify this.**

 This is possible due to the unique properties of the GCC panels. These panels incorporate nanosized luminescent glass nanocomposites, which not only convert sunlight to enhance red light for improved plant growth but also function as waveguides, similar to luminescent solar concentrators (LSCs). Since GCC panels can guide sunlight towards the silicon solar cells regardless of the direction of incoming light—whether under direct or indirect illumination—they help compensate for potential energy losses caused by panel tilting. By increasing the incoming photon flux to the silicon cells, the GCC panels minimize the impact of non-optimal panel angles and help maintain a high energy output. Clarify whether all test sites will use the same system layout as Figure 1 or if there will be variations. If differences exist, explain what aspects will be common or not common and how the sites will be compared.

**Quantitative results are missing all over. Include them**

 There will be variations in the system layout depending on the geographic location of the demonstration sites and the types of crops being cultivated. While the general concept remains the same, specific design parameters—such as the ratio of silicon panels to GCC panels—will be adjusted to optimize both energy output and crop growth under different climatic conditions. For example, since Türkiye and Spain receive higher annual sunlight levels, the proportion of silicon panels will be higher to maximize energy generation. In contrast, Denmark, with its lower sunlight levels, will have a higher GCC panel ratio to allow more light to reach the crops. The exact designs are still under investigation, and quantitative results will be provided once data collection begins at the demo sites. Comparative analysis will focus on key performance metrics, including energy yield, crop growth rates, and light distribution, to assess the effectiveness of different layouts across varying climatic conditions.

**Farming environment will be mostly dusty. What’s the plan to handle dust deposit?**

 Dust deposition is not a specific issue for our GCC panel design but rather a common challenge for all agrivoltaic systems. Mitigation strategies such as periodic cleaning, self-cleaning coatings, or optimized panel tilting to reduce dust accumulation can be considered, but addressing this challenge is beyond the specific scope of our design.

# Reviewer report: Reviewer response for version 1

**DOI:** 10.21956/openreseurope.20426.r49166

**Published:** 2025-01-16

**Authors:** Silvia Ma Lu

**Affiliations:** 1 Mälardalen University, Västerås, Sweden

The open letter titled “AgriPV system with climate, water and light spectrum control for safe, healthier and improved crop production” provides an overview of the objectives of the PV4Plants Horizon Europe project. The project aims to investigate the feasibility of combining agricultural production and solar energy conversion using perovskite-based PV panels optimized for light spectrum transmission. With three test sites across different countries, the project explores various crops, sustainability aspects, recyclability of systems, stakeholder engagement, financial models, and policy recommendations to advance AgriPV systems.

 The letter appears to primarily introduce the project’s plans rather than offering recommendations or policy implications. However, this is not fully clear, particularly given the inclusion of a “Results and analysis” section. Below are comments and suggestions to clarify and enhance the letter’s message.

 Responses to Peer-Review Form Questions:

 1. Is the rationale for the Open Letter provided in sufficient detail?

 Response: Partly.

 The challenges in agrivoltaics are outlined in the introduction, and the project’s objectives are clear. However, the letter’s purpose, whether to announce the project’s initiatives, encourage similar research, or discuss guidelines and policy was not clearly stated. Please, clarify this in the letter.

 2. Does the article adequately reference differing views and opinions?

 Response: Yes.

 The authors briefly mention perspectives from various stakeholders, such as farmers and policymakers.

 3. Are all factual statements correct, and are statements adequately supported by citations?

 Response: Partly.

 In general, statements in the letter lack citations (e.g., “agricultural sector [...] accounting for approximately 10% of all GHG emissions in the EU”, “tomato plants thrive under red light”, among others). Relevant references should be added at the end of each specific statement or group of statements, rather than grouping them all at the end of the section to make it easier for the reader to distinguish where each statement comes from.

 4. Is the Open Letter written in accessible language?

 Response: Yes.

 The language is clear, but some abbreviations (e.g., NGO) are unexplained, and others (e.g., PV) are defined multiple times. Additionally, the term "energy generation/production" could be replaced with "energy conversion" for accuracy.

 5. Are recommendations and next steps explained clearly for others to follow?

 Response: Not applicable.

 The letter does not explicitly offer recommendations or actionable next steps for the broader community. It primarily outlines project goals and future plans.

 Additional Comments and Suggestions:

- Structural improvements:
  The “System design, implementation, and testing” section is somewhat disorganized. The subsections could be reordered for clarity:
  - I suggest moving “Demonstration sites and testing” before “Multi-criteria assessment and financial models”. This way, the bullet points in "Advanced system design" and their apparent linked subsection are next to each other.
  - Elaborate on the bullet point about “Rainwater harvesting” to match the level of detail in the other bullet points which appear to have their own subsection “Real-time monitoring and proactive control” and “Material innovation and light spectrum optimisation”. How is the rainwater harvesting system done? Will it also be monitored?
- Demonstration sites and testing subsection:
  - Clarify whether all test sites will use the same system layout as Figure 1 or if there will be variations. If differences exist, explain what aspects will be common or not common and how the sites will be compared.
  - Provide details on stakeholder engagement and policy alignment methods, such as interviews, workshops, or surveys, to help readers understand how this part of the project will be carried out.
- Figure 3:
  - Suggestion to replace or complement this figure with an image or layout specific to the microalgae production AgriPV site, as this would better represent the content, similar to Figure 2.
- Results and analysis section:
  - According to Open Research Europe’s guidelines, open letters should not present new data or analysis. If this section includes unpublished results, the authors might consider submitting them as a research article instead.
  - If the results reference previous publications, add citations.
  - Those preliminary results involving laboratory tests or indoor trials seem to not be performed under the described AgriPV systems, kindly clarify their relevance and how the results can be translated to be used in the described open-field AgriPV systems. Else, I recommend to focus on expected outcomes (e.g., tools, guidelines, decision-making frameworks) and to emphasize what the community can gain from the project instead of experimental findings at this stage. This would perhaps align better with the open letter format.

Where applicable, are recommendations and next steps explained clearly for others to follow? (Please consider whether others in the research community would be able to implement guidelines or recommendations and/or constructively engage in the debate)

Not applicable

Does the article adequately reference differing views and opinions?

Yes

Are all factual statements correct, and are statements and arguments made adequately supported by citations?

Partly

Is the rationale for the Open Letter provided in sufficient detail? (Please consider whether existing challenges in the field are outlined clearly and whether the purpose of the letter is explained)

Partly

Is the Open Letter written in accessible language? (Please consider whether all subject-specific terms, concepts and abbreviations are explained)

Yes

Reviewer Expertise:

Agrivoltaics, Solar Energy, Spectral Irradiance, Wavelength-Selective Solar Photovoltaic

I confirm that I have read this submission and believe that I have an appropriate level of expertise to confirm that it is of an acceptable scientific standard, however I have significant reservations, as outlined above.
